# Phylogenomics of *Leptospira santarosai*, a prevalent pathogenic species in the Americas

**DOI:** 10.1371/journal.pntd.0011733

**Published:** 2023-11-02

**Authors:** Diana Chinchilla, Cecilia Nieves, Ricardo Gutiérrez, Vallier Sordoillet, Frédéric J. Veyrier, Mathieu Picardeau

**Affiliations:** 1 Centro Nacional de Referencia de Bacteriología, Instituto Costarricense de Investigación y Enseñanza en Nutrición y Salud (INCIENSA), La Unión, Cartago, Costa Rica; 2 Bacterial Symbionts Evolution, Centre Armand-Frappier Santé Biotechnologie, Institut National de la Recherche Scientifique, Université du Québec, Laval, Québec, Canada; 3 Institut Pasteur, Université Paris Cité, Biology of Spirochetes Unit, Paris, France; Yale University School of Medicine, UNITED STATES

## Abstract

**Background:**

Leptospirosis is a complex zoonotic disease mostly caused by a group of eight pathogenic species (*L. interrogans*, *L. borgpetersenii*, L. *kirschneri*, *L. mayottensis*, *L. noguchii*, *L. santarosai*, *L. weilii*, *L. alexanderi*), with a wide spectrum of animal reservoirs and patient outcomes. *Leptospira interrogans* is considered as the leading causative agent of leptospirosis worldwide and it is the most studied species. However, the genomic features and phylogeography of other *Leptospira* pathogenic species remain to be determined.

**Methodology/principal findings:**

Here we investigated the genome diversity of the main pathogenic *Leptospira* species based on a collection of 914 genomes from strains isolated around the world. Genome analyses revealed species-specific genome size and GC content, and an open pangenome in the pathogenic species, except for *L. mayottensis*. Taking advantage of a new set of genomes of *L. santarosai* strains isolated from patients in Costa Rica, we took a closer look at this species. *L. santarosai* strains are largely distributed in America, including the Caribbean islands, with over 96% of the available genomes originating from this continent. Phylogenetic analysis showed high genetic diversity within *L. santarosai*, and the clonal groups identified by cgMLST were strongly associated with geographical areas. Serotype identification based on serogrouping and/or analysis of the *O*-antigen biosynthesis gene loci further confirmed the great diversity of strains within the species.

**Conclusions/significance:**

In conclusion, we report a comprehensive genome analysis of pathogenic *Leptospira* species with a focus on *L. santarosai*. Our study sheds new light onto the genomic diversity, evolutionary history, and epidemiology of leptospirosis in America and globally. Our findings also expand our knowledge of the genes driving *O*-antigen diversity. In addition, our work provides a framework for understanding the virulence and spread of *L. santarosai* and for improving its surveillance in both humans and animals.

## Author summary

Leptospirosis is an emerging zoonosis caused by pathogenic species of a highly heterogeneous genus. Most studies have focused on *Leptospira interrogans* that is responsible for the majority of human infection cases worldwide. On the contrary, our knowledge is very limited for other pathogenic species, including *L. santarosai*, which may represent a public health problem in both humans and animals in the American continent. Our comparative genomic analyses of the pathogenic species revealed that *L. santarosai* is characterized by an open pangenome state with high genetic and serovar diversity. This first study of *L. santarosai* isolates not only contributes to the global understanding of genomics and evolution within the *Leptospira* pathogenic species but also provides the groundwork for better surveillance of this pathogen.

## Introduction

*Leptospira* is a highly heterogeneous bacterial genus divided into pathogenic and saprophytic species and then further divided into more than 300 serovars, which are defined according to structural heterogeneity of the lipopolysaccharide (LPS) *O*-antigen. Nowadays, strain identification is mainly based on genome analysis, and core genome multilocus sequence typing (cgMLST) [1] enables identification of the species and below. Recent studies have also shown that whole-genome sequences can be used for predicting *Leptospira* serotypes on the basis of the *rfb* locus which contains the genes for the *O*-antigen biosynthesis [2,3]. This approach offers a promising alternative to the conventional serotyping method, which is laborious, time-consuming, expensive and requires a high level of expertise.

Over the past decade, the number of *Leptospira* species described has rapidly extended from 22 in 2014 to 69 in 2022 [4], largely due to the use of improved protocols for culture isolation from the environment [5,6] and the generalization of next generation sequencing [7]. Among the genus *Leptospira*, eight species (*L. interrogans*, *L. kirschneri*, *L. noguchii*, *L. santarosai*, *L. mayottensis*, *L. borgpetersenii*, *L. alexanderi* and *L. weilii*), which diverged after a specific node of evolution, constitute the most virulent group of pathogenic species [8]. These *Leptospira* species are the causative agents of leptospirosis in both human and animals, leading to a high disease burden in tropical countries [9] and major economic losses in the livestock sector [10].

Our previous analysis of the distribution of pathogenic *Leptospira* species showed that *L. interrogans* is the most frequently encountered and globally distributed species [1]. This cosmopolitan species is also by far the most studied in terms of virulence, and molecular epidemiology, among other aspects. On the contrary, to date, very little is known about the geographical distribution, reservoirs, genomic features and virulence factors of pathogenic species other than *L. interrogans*. In the same analysis, we showed that some pathogenic species were geographically restricted [1]. Thus, only limited reports have described the existence of *L. santarosai* outside the American continent. *L. santarosai*, named after Carlos A. Santa Rosa, a Brazilian veterinary microbiologist who pioneered the study of leptospirosis in Brazil, was first described in 1987 [11]. *L. santarosai* is predominant in many countries from Central and South America.

In the present study, we first performed an analysis of the pangenome in pathogenic *Leptospira* species and then took a closer look at the genetic diversity of *L. santarosai* including a set of strains recently isolated from patients in Costa Rica, which is an endemic country for leptospirosis [12].

Phylogenomics analysis of *L. santarosai* genomes will enable to better understand the genetic diversity and genome features of this pathogenic species which is prevalent in most countries of the American continent.

## Material and methods

### Ethics statement

According to the decree number 40556-s of the General Health Law of Costa Rica, epidemiological studies that incorporate the review of clinical records do not require the approval of an ethics-scientific committee. Additionally, no written informed consent from patients was required, as the study was conducted as part of the routine diagnosis at the Centro Nacional de Referencia de Bacteriología of the Instituto Costarricense de Investigación y Enseñanza en Nutrición y Salud (INCIENSA). No additional clinical specimens were collected for the purpose of the study. Human samples were anonymized, and collection of the samples was conducted according to the Declaration of Helsinki.

### Strains

Isolates sequenced in this study (n = 153) were obtained from the collections of the French National Reference Center for Leptospirosis (Institut Pasteur, Paris, France), Laboratorio de Genética Molecular (Instituto Venezolano de Investigaciones Científica, Caracas, Venezuela), Institut Pasteur of Alger (Algiers, Algeria), Institute of Veterinary Bacteriology (University of Bern, Switzerland), Molecular Epidemiology and Public Health Laboratory (School of Veterinary Sciences, Massey University, New Zealand), Instituto de Higiene (Facultad de Medicina, Universidad de la República, Montevideo, Uruguay), Universidade Federal Fluminense (Rio de Janeiro, Brazil), Faculty of Veterinary Medicine (University of Zagreb, Croatia), National Collaborating Centre for Reference and Research on Leptospirosis (Academic Medical Center, Amsterdam, the Netherlands), Laboratory of Zoonoses (Pasteur Institute in Saint Petersburg, Saint Petersburg, Russia), Institute for Medical Research (Malaysia), Faculty of Medicine and Health Sciences (University Putra Malaysia, Malaysia), and Leptospirosis Research and Expertise Unit (Institut Pasteur Nouvelle-Calédonie, Nouméa, New Caledonia), Kimron Veterinary Institute (Israel). We also downloaded genomes from our previous studies including isolates from the collections of Lao-Oxford-Mahosot Hospital-Wellcome Trust-Research Unit (LOMWRU) (Microbiology Laboratory, Mahosot Hospital, Vientiane, Lao People’s Democratic Republic), Unidad Mixta Pasteur-Instituto Nacional de Investigación Agropecuaria (Institut Pasteur of Montevideo, Montevideo, Uruguay), Centre Hospitalier de Mayotte (France), and Department of Mycology-Bacteriology (Institute of Tropical Medicine Pedro Kourí, Havana, Cuba) [1,2,13–15] as well as genomes from the NCBI database. Information on strains and genomes used in this study are indicated in **S1 and S2 Tables**.

### Whole-genome sequencing

Illumina sequencing was performed from extracted genomic DNAs of exponential-phase cultures using a MagNA Pure 96 Instrument (Roche, Meylan, France). Next-generation sequencing (NGS) was performed using Nextera XT DNA Library Preparation kit and the NextSeq 500 sequencing systems (Illumina, San Diego, CA, USA) at the Mutualized Platform for Microbiology (P2M) at Institut Pasteur. CLC Genomics Workbench 9 software (Qiagen, Hilden, Germany) was used for analyses. The generated contig sequences together with the sample metadata are available in BIGSdb hosted at the Institut Pasteur (https://bigsdb.pasteur.fr/leptospira/). We also downloaded additional genome sequences of *Leptospira* isolates from the NCBI database (**S1 Table**). Only genomes meeting quality requirements, such as i) sequencing coverage >30x, ii) number of contigs <600, iii) cumulative contigs length within the typical range of *Leptospira* genomes (3.6-6Mb), iv) GC content within the typical range of *Leptospira* genomes (35–48%), and v) <100 uncalled cgMLST alleles out of the 545 pre-defined core genes, were selected for further analyses.

### Genomic analyses

Comparative analyses of the pangenome were performed using two software: Roary version 3.11.2 [16], and a combination of COG and OMCL algorithms in GET_HOMOLOGUES version 20190411 [12]. Both methods yielded a similar number of gene clusters. In the Roary analysis, a 60% identity cut-off was applied to define gene clusters (option -i 60), and no other parameters were modified. Among the Roary outputs, a tab-separated file containing the number of genes in the pangenome was used to create a graph depicting the variation in the number of gene clusters as a function of the number of genomes analyzed. Roary iterated 10 times, calculating the number of new genes added as each genome was sequentially incorporated into the analysis. This graph facilitated a quick determination of whether the pangenome was open or closed and allowed for the calculation of the α coefficient in Heap’s Law (n = κNγ, with γ = 1- α) [17]. On the other hand, GET_HOMOLOGUES was used to infer the pangenome distribution in cloud-, shell-, soft-core-, and core-genome. This was achieved by generating a tab-separated pangenome matrix file that included the number of all the clusters identified by both COG and OMCL algorithms. The matrix represented the intersection of the two methods and served as input for the parse_pangenome_matrix.pl script within GET_HOMOLOGUES, which classified the clusters as cloud (shared by up to 2 genomes), shell (shared by more than 2 genomes but less than 93% of genomes analyzed), soft-core (shared by 93–99% of genomes), or core-genes (shared by 100% of genomes). Due to the substantial number of genomes available for *L. interrogans* and *L. borgpetersenii*, as well as the redundancies observed in serogroups and serovars, representative genomes of each serogroup/serovar were selectively chosen to mitigate computational costs. Excluding genomes with redundant identities is not anticipated to result in significant alterations in the pangenome distribution.

Genome size and GC content for highly virulent *Leptospira* species were determined through DFAST annotation [18]. Individual values were plotted and grouped per species, with the mean and standard deviation displayed. Genome size and GC content were compared using the Kruskal-Wallis Rank Sum Test, for the comparison of *Leptospira* spp. and the Wilcoxon rank test, for the comparison of two phylogenetic-related groups. Post-hoc comparisons were performed using Dunn’s Kruskal-Wallis Multiple Comparisons (Dunn, 1964). P-values were adjusted with the Bonferroni method. Statistical analyses were performed in R [19], using FSA package [20].

Average Nucleotide Identity (ANI) and Percentage of Conserved Proteins (POCP) were calculated for the 64 *L. santarosai* genome sequences as well as *L. interrogans* str. Fiocruz L1-130 and *L. borgpetersenii* str. M84 used as outgroups (S1 and S2 Figs). Genomes were annotated by Prokka version 1.13.7 [21]. ANI and POCP matrices were inferred using OMCL algorithm via GET_HOMOLOGUES version 20190411 [12]. Briefly, to calculate ANI, the option -A was employed along with option -a to utilize nucleotide sequences and perform BLASTN. This process generated a tab-separated file containing average percentage sequence identity values between pairs of genomes, calculated from sequences within all identified clusters (option t = 0). This tab-separated file served as the input to create a symmetric matrix, where the genomes were clustered based on their ANI values. Dendrograms based on this clustering were generated on both sides of the matrix to visually represent the proximity among genomes. Similarly, POCP was calculated by including the option -P and performing default BLASTP searches. This step yielded another tab-separated file, which was subsequently used to create a symmetric matrix. Analogous to the ANI matrix, the genomes were clustered based on the shared % of conserved proteins between pairs of genomes. These values are calculated as POCP = (C_a_ + C_b_)/(total_a_ + total_b_), where C_a_ and C_b_ denote the number of conserved proteins from genome a in genome b and from genome b in genome a, respectively, normalized by the sum of total proteins in each genome. The clustering process also generated dendrograms, indicating the proximity among genomes in terms of conserved proteins.

Core genome MLST (cgMLST) typing was performed using a scheme based on 545 core genes as previously described [1]. *L. santarosai* core-genome based phylogeny was constructed using the 1288 core-genes alignment resulting from Roary analysis (60% identity cut-off, option -i 60). The best-fit model and the maximum-likelihood phylogenetic tree were determined by IQ-TREE version 1.6.11 [22], considering 10,000 ultrafast bootstraps [23]. *L. interrogans* str Fiocruz L1-130 and *L. borgpetersenii* str. M84 were used as outgroups. Tree branches were transformed with the "proportional" option on FigTree software v1.4.4 (http://tree.bio.ed.ac.uk/software/figtree/), which adjusts branch distances according to the number of tips under each node to improve visualization of the tree. Gene presence/absence analyses among *rfb* clusters from different genomes here studied were performed by protein-level searches using BLASTP [24] and subsequent network associations by NetworkX version 2.6.2 [25]. A similarity threshold of 60% was applied, as previously described [2]. The resulting presence/absence table obtained from the network association analysis was converted into a binary CSV file, where 0 represents gene absence and 1 represents gene presence. This binary table was subjected to hierarchical clustering based on shared protein-encoding genes (options: euclidean distance, ward linkage) using available tools at https://mev.tm4.org. Jaccard’s similarity index was used to measure the similarity between *rfb* patterns.

### Genomic data

The sequencing data generated in this study are available in the NCBI database under the BioSample accession numbers SAMN34670613, SAMN34670614, SAMN34670615, SAMN34670616, SAMN34670617, SAMN34670618, SAMN34670619, SAMN34670620, SAMN34670621, SAMN34670622, SAMN34670623, SAMN34670624, SAMN34670625, SAMN34670626, SAMN34670627, SAMN34670628, SAMN34670629, SAMN34670630. Genome sequences used in this study are also available at https://bigsdb.pasteur.fr/cgi-bin/bigsdb/bigsdb.pl?db=pubmlst_leptospira_isolates&page=query&project_list=21&submit=1.

## Results and discussion

### Distribution of pathogenic Leptospira species shows that L. santarosai isolates are mostly from the Americas

We first investigated the geographical distribution of pathogenic *Leptospira* species using 914 genomes of isolates collected between 1928 and 2022 (**S1 Table**).

**Fig 1:**
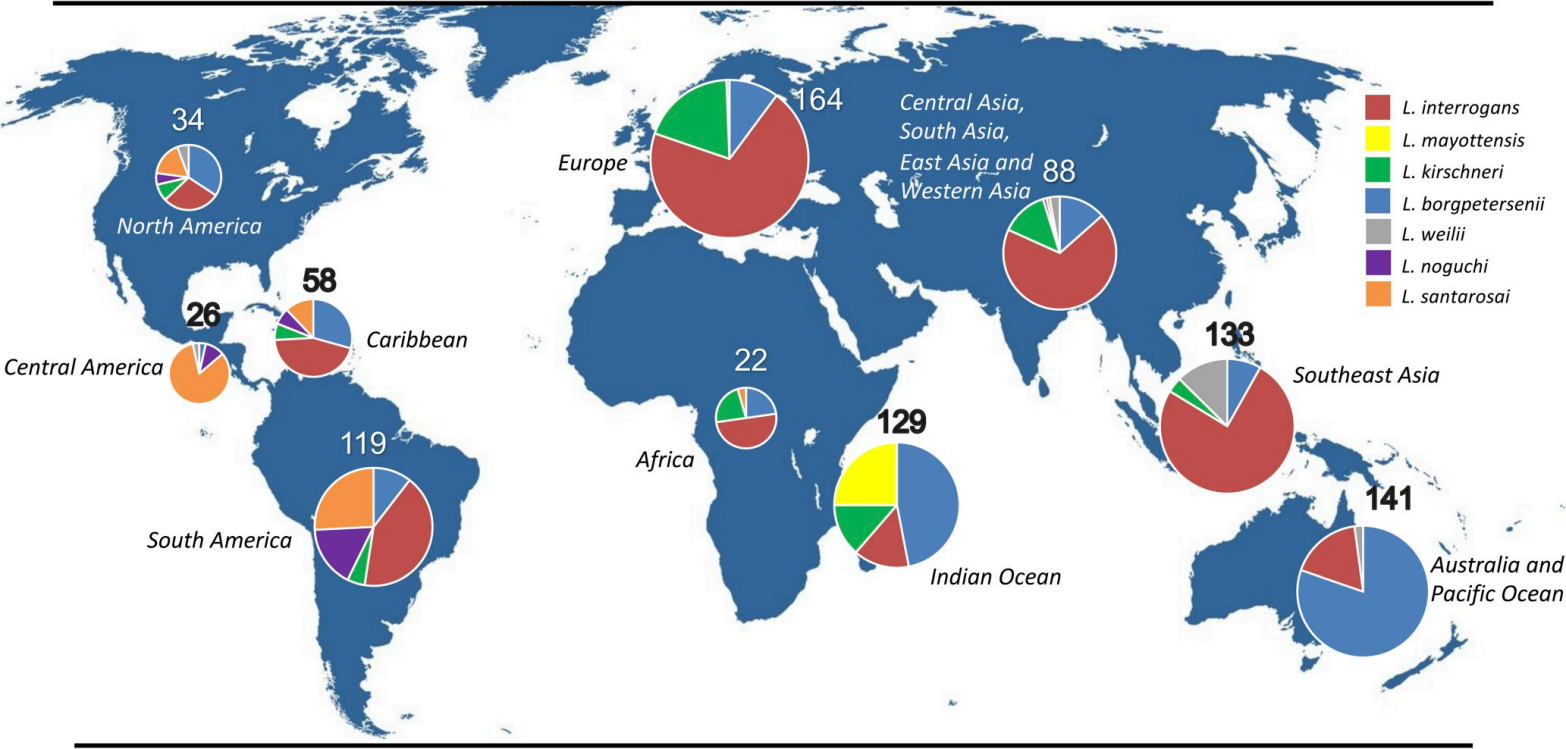
Geographic origins of the most frequent pathogenic *Leptospira* species in our genome database (n = 914). Each pie chart corresponds to a given world region. As shown in our map, *L. santarosai* (n = 64) is mostly found in America (North America, Central America, South America and the Caribbean islands). The base layer of the map is freely available from outline-world-map.com.

Species included in our study are: *L. interrogans* (n = 410), *L. borgpetersenii* (n = 264), *L. kirschneri* (n = 88), *L. mayottensis* (n = 33), *L. noguchii* (n = 31), *L. santarosai* (n = 64), *L. weilii* (n = 24); *L. alexanderi*, with only 2 isolates in our database, was not included in this study. Strains were isolated from human (50%) and animal (49%) samples, in Europe (18 %), Africa (2 %), Indian Ocean (14%), Caribbean islands (6%), Central America (3%), South America (13%), North America (4%), Central Asia, South Asia, East and Western Asia (11%), Southeast Asia (14 %), and Australia and the Pacific region (15%) (**Fig 1**). Although this study is based on the genomes available in the databases and may introduce a bias, *L. interrogans*, *L. kirschneri*, and *L. borgpetersenii* are distributed worldwide, *L. weilii* is mostly found in Asia, Australia and the Pacific region, *L. mayottensis* in the Indian Ocean, and *L. noguchii* and *L. santarosai* in America as previously shown [1].

Leptospirosis is endemic in most countries of South and Central America, as well as in the Caribbean region [9,26–28]. In addition, most outbreaks of leptospirosis have been reported in the Latin America and the Caribbean region [29], where the disease is widespread in domestic and wild animals [12]. However, comprehensive data concerning human and animal leptospirosis remain largely scarce in most American countries [30]. We previously studied the genomes of *L. noguchii* isolated from human and animals in America [2] but our knowledge of *L. santarosai*, the other prevalent species in America, is rather limited.

Here, we sequenced 18 *L. santarosai* strains, including twelve strains that were isolated in Costa Rica in 2020–2021 from patients. The ANI and POCP values were calculated for the 64 *L. santarosai* strains further confirming they all belong to the same species (S1 and S2 Figs). Of the 64 *L. santarosai* strains in our genome database, 28 were isolated in South America (Brazil, Colombia, Ecuador, Peru), 21 in Central America (Costa Rica, Panama), 6 in North America (US; not including Puerto Rico), 7 in the Caribbean region (Martinique, Guadeloupe, Trinidad and Tobago and Puerto Rico), and only two strains were isolated outside the Americas (China and Democratic Republic of the Congo) (**Fig 1** and **S2 Table**). Of note, *L. santarosai* has not been isolated in Uruguay, where a large number of *Leptospira* strains have been isolated from cattle [15].

*L. santarosai* strains in our study were isolated from humans (n = 38), bovine (n = 12), rodents (n = 8, including rats, spiny rats, capybara and muskrat), opossum (n = 2), dog (n = 1), goat (n = 1), pig (n = 1), and racoon (n = 1) (**S2 Table**).

Previous studies have shown that *L. santarosai* can be detected from different sources in many countries of America and the Caribbean region. It is the predominant species in humans, rodents and dogs in Peru and Colombia [31,32]. In Peru, it has additionally been found in rural environmental water samples (but not in urban samples), as well as in association with pigs and cattle [33]. In Brazil, *L. santarosai* has been isolated from dogs [34], cattle [35], goats [36], and capybaras [37]. Moreover, it has also been identified in patients in French Guiana [38], Guadeloupe [39] and the US [40].

Only a few reports have described the existence of *L. santarosai* outside the American continent. Some years after the original description of *L. santarosai* [11], Brenner *et al*. listed 65 *L. santarosai* strains, of which only three were isolated from outside America [41]. One *L. santarosai* strain was isolated from a patient in Sri-Lanka in 1966 but has never been reported in this country afterwards [42, 43]. The other two strains were isolated in Denmark and Indonesia but, again, *L. santarosai* has not been subsequently isolated in these countries. More recently, a strain belonging to *L. santarosai* serogroup Grippotyphosa was isolated from a patient in India and its genome sequenced [44]. However, because of highly fragmented genome (884 contigs) and missing genomic data (135 uncalled cgMLST alleles), the cluster assignment was not possible for this isolate and we removed its genome from our analysis.

The serogroup Shermani, is commonly reported in serological surveys in animals in Asia [45–48]. Unfortunately, there is no evidence that the infecting strains described in these studies were *L. santarosai* or another species such as *L. noguchii* and *L. inadai* which also contain serovars from the serogroup Shermani [49]. Finally, the other country outside Americas where *L. santarosai* was reported is Taiwan in East Asia. Serogroup Shermani, presumably belonging to *L. santarosai*, is predominant among patients with severe leptospirosis in Taiwan [50]. However, only one *L. santarosai* strain, strain CCF, has been isolated from a patient with leptospirosis in Taiwan [51] and this strain, for which we do not have the complete genome [52], is no longer available (personal communication of Prof Chih-Wei Yang).

### Genome analysis shows species-specific features and an open pangenome for most pathogenic Leptospira species

**Fig 2:**
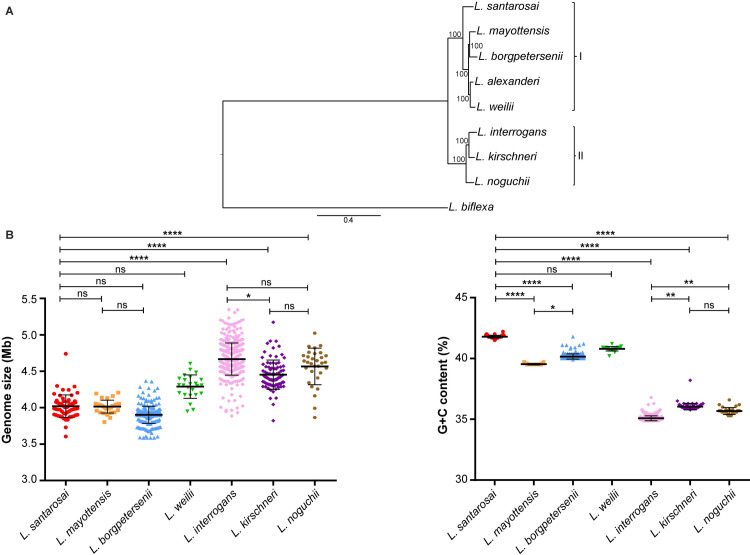
Genome characteristics of pathogenic *Leptospira*. A. Phylogenetic tree built using IQTREE on the alignment of 645 soft-core genes (60% identity cut-off, present in at least 95% of the genomes). The bootstraps values are indicated in the tree (as a percentage). B. The variation of size and G+C% are indicated for *L. interrogans* (n = 410), *L. borgpetersenii* (n = 264), *L. kirschneri* (n = 88), *L. mayottensis* (n = 33), *L. noguchi* (n = 31), *L. santarosai* (n = 64) and *L. weillii* (n = 24); *L. alexanderi*, with only 2 isolates in our database, was not included in this study. Statistical differences between *L. santarosai* and other pathogenic species were determined with the non-parametric ANOVA test (Kruskal-Wallis).

Phylogenetic analysis of the eight pathogenic *Leptospira* species using the saprophyte *L. biflexa* as the outgroup shows two distinct groups as previously shown [8, 53]. One phylogenetic group constituted by *L. santarosai*, *L. mayottensis*, *L. borgpetersenii*, *L. alexanderi* and *L. weilii* (group I), and another one with *L. interrogans*, *L. kirschneri* and *L. noguchii* (group II) (**Fig 2A**). The genome size and GC content vary widely among pathogenic species usually correlating with these two phylogenetic subgroups (**Fig 2B and S3 and S4 Tables**). Notably, the genome size of the group I (3.96 ± 0.17 Mb) is significantly smaller to species from the group II (4.63 ± 0.32 Mb; Wilcoxon rank test, W = 4985, *p*< 2.2e-16; **Fig 2B**, left panel). Inversely, guanine+cytosine content (G+C%) is higher in the group I (40.4 ± 0.7 G+C%) than the group II (35.5 ± 0.43%; Wilcoxon rank test, W = 207668, *p*< 2.2e-16; **Fig 2B**, right panel**)**. Interestingly, with the exclusion of *L. weilii*, the genome sizes were not significantly different between species of the group I (Dunn´s test, all pair comparisons with *p* adjusted >0.05), supporting their characterization as a monophyletic group. However, the G+C% content did differ significantly between *Leptospira* spp. within group I. (Dunn´s test, all pair comparisons with *p* adjusted value ≤0.04). Variation was larger within the species of group II, as *L. interrogans* shows a larger genome size than *L. kirschneri* (Dunn´s test, *p* adjusted ≤0.0000003), whereas G+C% was significantly different between *L. interrogans* and *L. kirschneri*, and *L. interrogans* and *L. noguchii* (Dunn´s test, all pair comparisons with *p* adjusted value ≤0.0002). These disparities in GC content and genome size may be a response to long-term niche adaptation of pathogens which emerged hundred million years ago at the same time as the appearance of mammals [54].

**Fig 3:**
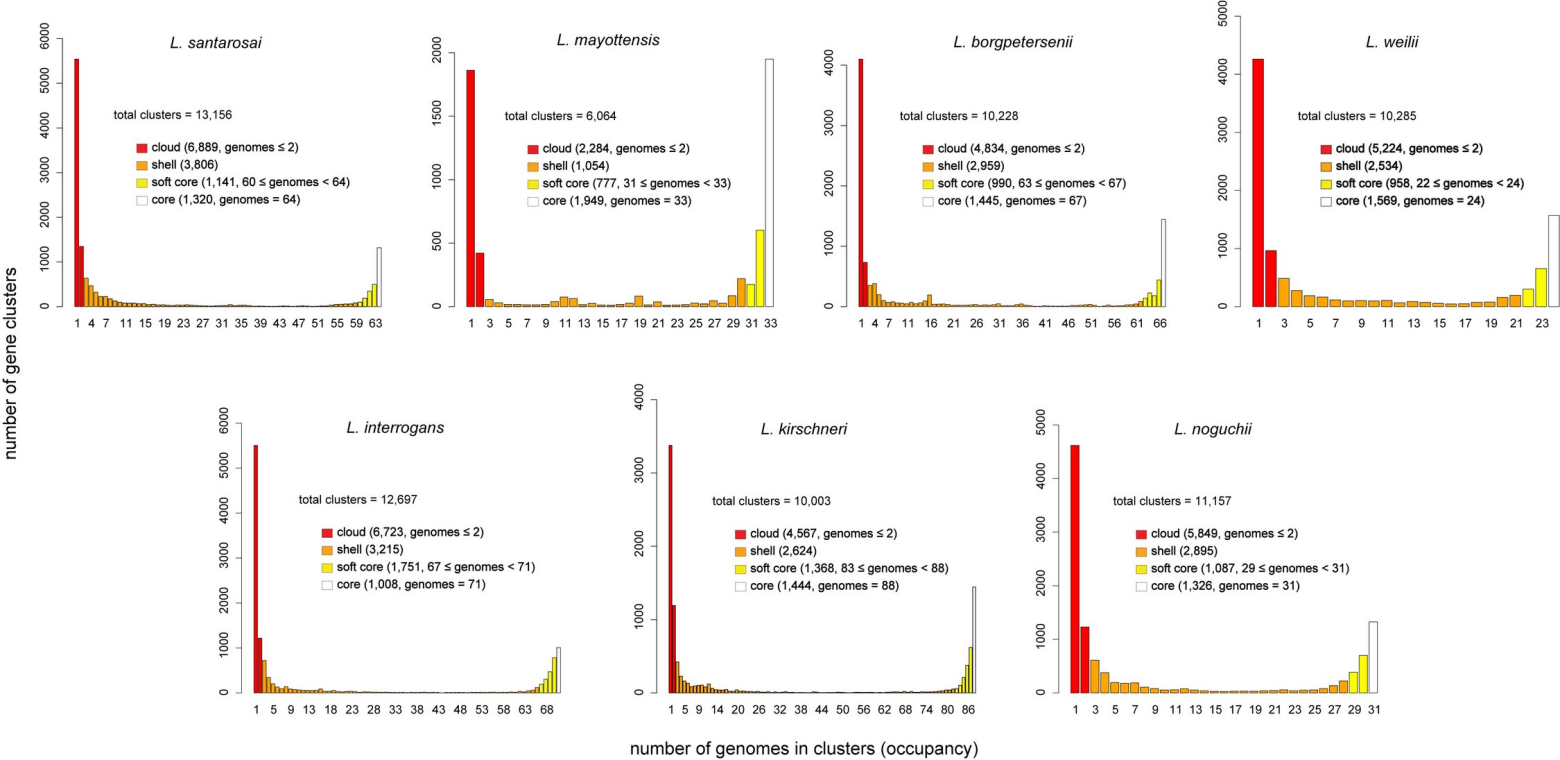
Pangenome distribution in four categories (cloud, shell, soft core and core) for *Leptospira* pathogenic species. Analyses done with GET_HOMOLOGUES showing the U-shaped distribution of pangenome from *L. santarosai*, *L. mayottensis*, *L. borgpetersenii*, *L. weilii*, *L. interrogans*, *L. kirschneri* and *L. noguchii*. For *L. interrogans* and *L. borgpetersenii*, a subset of representative genomes of all sizes and from all geographic locations was selected to reduce computational costs and avoid redundancy.

Most pathogenic species exhibit a strong enrichment (>3X) of accessory genes (**Fig 3**). However, it is important to notice that *L. mayottensis* stands as an exception, where the analysis of 33 strains showed a distribution of 1,949 and 2,284 genes constituting the core and the accessory genomes, respectively (1.2X enrichment), which probably resides in the fact that *L. mayottensis* is restricted to the Indian Ocean (Mayotte and Madagascar) and its specific adaptation to tenrec, described as the main reservoir of this pathogenic species [55,56].

Concerning specifically *L. santarosai*, it harbors an open pangenome suggesting a great diversity in its gene repertoire (**S3 Fig**). Our analysis shows a strong enrichment (≈5X) of gene clusters that are unique to a maximum of two genomes (6,889) compared to core genome gene clusters (1,320) (**Fig 3**).

### High genetic diversity of L. santarosai strains

**Fig 4:**
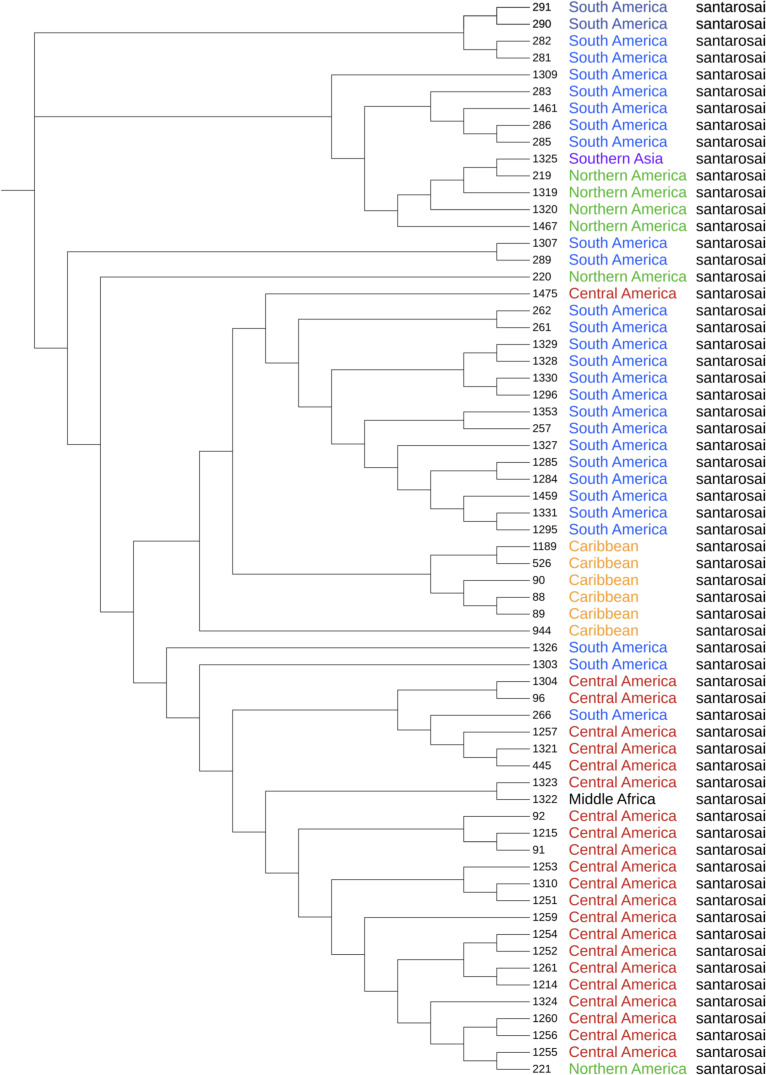
Phylogenetic tree of *L. santarosai* strains. Maximum-likelihood phylogeny based on the variable sites of the cgMLST scheme consisting of 545 core genes showing the distribution of species, serogroups and geographic origins. The species *L. santarosai* (n = 64) were divided into 55 cgMLST clonal groups belonging to 9 different serogroups; for 20 strains the serogroup is unknown or undetermined. Colors indicate strains isolated from the same geographic region (Central America in red, South America in blue, North America in green, Caribbean in orange, Southern Asia in purple, and Middle Africa in black). Branch lengths were not used to ease readability of groups and isolates.

To further investigate the genetic diversity of *L. santarosai* isolates, we used a core genome MLST (cgMLST) scheme [1] (**Fig 4**). The species *L. santarosai* (n = 64) were divided into 55 cgMLST clonal groups (cgCGs) showing a high intraspecies genetic diversity (**Fig 4**) as shown in previous studies [29,32,39,57,58]. Among the 55 cgCGs, none is composed of more than 3 strains (**S1 Table**), and none is composed of both human and animal strains. We cannot therefore identify transmission of *L. santarosai* clones between different hosts. There is a wide range of possible reservoirs for *L. santarosai* in the Americas. Some countries in the region are among the largest cattle producers in the world so these animals could be important reservoirs for human infections. The Americas also exhibit a great biodiversity, so many species of wild animals such as rodents, marsupials, and domestic animals, such as dogs may be involved in transmission cycles. Among the 15 strains isolated from patients in Costa Rica, only two (id1256 and id1260) exhibit the same clonal group further confirming the great diversity of strains even within one small country.

Unfortunately, analyses to identify associations of *Leptospira* genotypes to particular epidemiological variables (host reservoir, disease outcome, etc.) cannot be performed with our small sample size. However, we could determine some phylogeographic lineages. A clear geographical separation of the clonal groups was observed for strains from (i) Central America, comprising isolates from Costa Rica (15; all human strains), Panama (6) but also one strain from South America (Colombia); South America which was further divided in two divergent groups, (ii) one containing strains from Brazil (10; mostly bovine strains) and the other (iii) including strains from Peru (9); and (iv) Caribbean islands, with strains from Guadeloupe (2), Martinique (2), Trinidad (1) and Puerto Rico (1) (**Fig 4**). This suggests ancestral presence of this species in these different countries and further separated evolution with no or low geographic diffusion. On the contrary, previous phylogenetic analyses of *L. noguchii* [2] and *L. interrogans* [1] did not reveal a correlation of genotype with geographical distribution.

### Diversity of serovar and O-antigen-encoding locus in L. santarosai

Further identification of *L. santarosai* strains was performed at the level of serovar and serogroup. Serogroups Grippotyphosa (8), Shermani (6), Tarassovi (8), Mini (5), Javanica (3), Pyrogenes (7), Sejroe (3), Hebdomadis (2), and Sarmin (2) were identified by serogrouping, but serovar/serogroup could not be assigned for 20 strains (no agglutination in serogrouping analyses or absence of serotyping information in the genome database). The three strains of the Javanica serogroup have been identified as belonging to a new serovar, Arenal, circulating in patients in Costa Rica [59,60].

**Fig 5:**
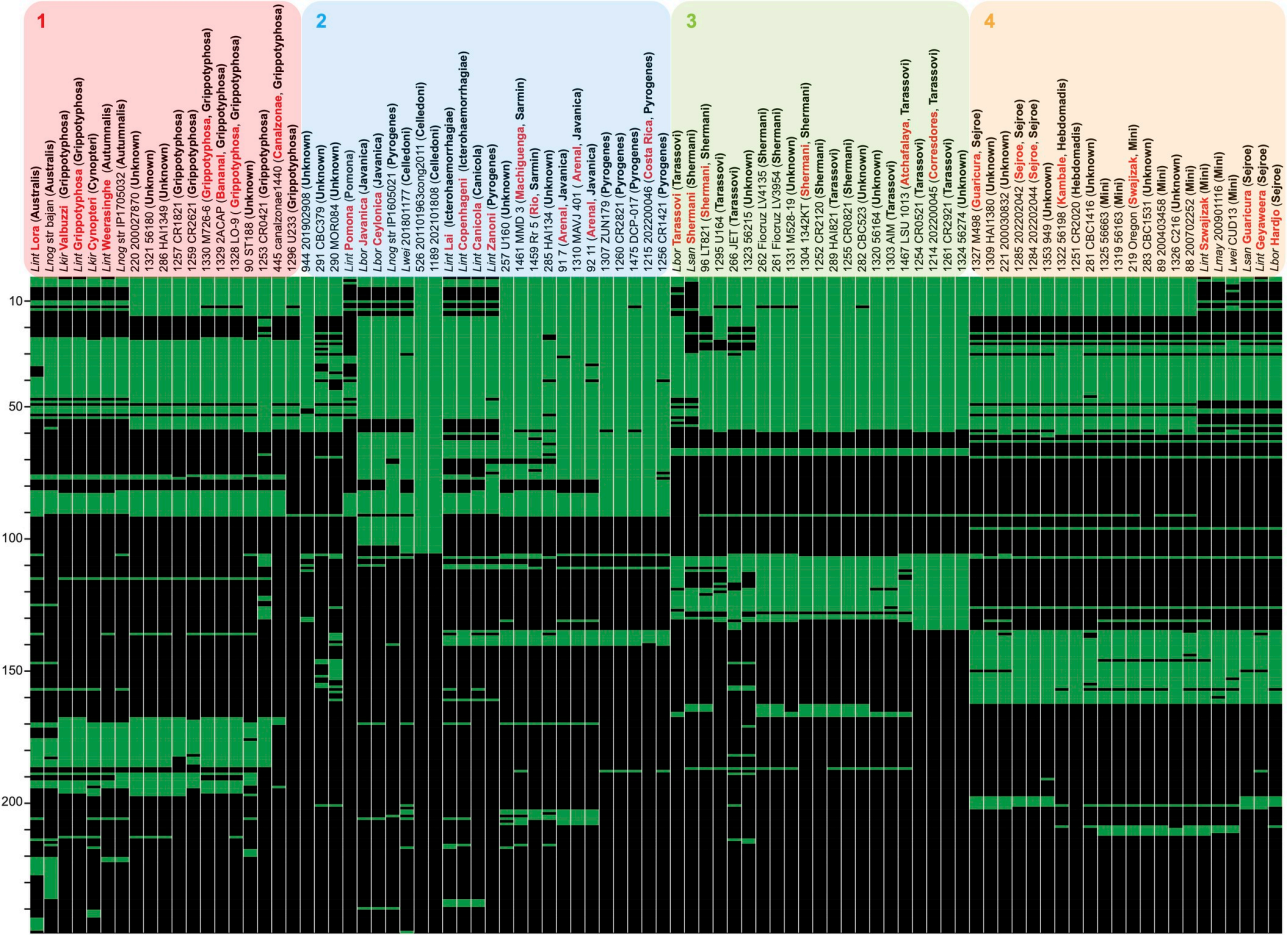
Gene presence/absence matrix of *rfb* clusters from different *Leptospira* strains and species, covering a range of distinct serogroup/serovar identities compared to *L. santarosai* strains. Horizontal lines correspond to individual genes or set of genes grouped according to their percentage of similarity (cut-off 60%), green meaning presence, and black absence. Scales on the left of the matrices indicate the number of different genes being compared. Columns correspond to different *Leptospira* strains as indicated on the columns’ labels. Those whose name begins with the species, correspond to the *rfb* cluster of known serovar/serogroup strains (serovar indicated in red, serogroup in brackets and in bold). Names of the *L. santarosai* strains analyzed in the present study begin with their ID number followed by the name of the strain. In brackets, serovar is indicated in red, if assigned, and serogroup in bold. For the latter strains, the whole genome was used and compared against the other *rfb* and a pan-*rfb* reference. Strains were organized after hierarchical clustering considering presence/absence of *rfb* genes. Four major clusters can be evidenced: 1) including serogroups Australis, Grippotyphosa, Cynopteri, and Autumnalis; 2) the most variable, comprising serogroups Tarassovi, Pomona, Javanica, Pyrogenes, Celledoni, Icterohaemorrhagiae, Canicola and Sarmin; 3) composed only of serogroups Tarassovi and Shermani; and 4) including serogroups Sejroe, Hebdomadis and Mini.

We recently showed that the analysis of the gene content of the LPS *O*-antigen–encoding cluster, or *rfb* cluster, correlates with *Leptospira* serovar and serogroup identity [2]. We then analyzed the gene composition of the *rfb* cluster of our set of *L. santarosai* strains, including 20 strains of unknown serogroup, in comparison to the *rfb* from reference strains of known serovars/serogroups (**Fig 5**). Strains were organized after hierarchical clustering considering presence/absence of the *rfb* genes. As previously shown, well-typed reference serovars from the same serogroup share the same patterns [2] but serovars from different serogroups may also share a similar genetic fingerprint: this is the case, for example, between serovars Copenhageni, Lai (both from serogroup Icterohaemorrhagiae) and serovar Canicola (serogroup Canicola), or serovars belonging to serogroups Grippotyphosa, Cynopteri and Autumnalis or between serovars from serogroups Mini, Sejroe and Hebdomadis. Four major clusters of genetic fingerprints can be distinguished (**Fig 5**) and were further confirmed using Jaccard’s similarity index (**S4 Fig**): cluster 1, containing reference strains from serogroups Australis, Grippotyphosa, Cynopteri and Autumnalis; cluster 2, which includes more variability of genetic fingerprints with reference strains from serogroups Pomona, Javanica, Pyrogenes, Celledoni, Icterohaemorrhagiae, Canicola and Sarmin; cluster 3, composed only of reference strains from serogroups Tarassovi and Shermani; and cluster 4, that comprises reference strains from serogroups Sejroe, Hebdomadis and Mini (**Fig 5**). The 64 *L. santarosai* strains were then assigned to the different cluster according to *rfb* gene composition (**S1 Table**). Cluster 1 contains all *L. santarosai* strains identified as belonging to serogroup Grippotyphosa (id1259, id1257, id1296, id445, id1328, id1329, id1330, id1253), as well as four strains of unknown serogroups (id220, id1321, id286, id90) showing patterns related to Grippotyphosa. Interestingly, id220, id1321 and id286 were isolated at three different geographic locations: North (id220), Central (id1321) and South America (id286). The three strains are phylogenetically distant, but exhibit an identical *rfb* cluster gene composition, suggesting that the *rfb* genomic island, important for bacterial persistence and adaptation to specific reservoir hosts, has disseminated in unrelated *L. santarosai* strains. Strains from the serogroups Sarmin (id1459, id1461), Javanica (id91, id1310, id92; all from the new serovar Arenal recently described in Costa Rica), Pyrogenes (id1307, id1260, id1475, id1215, id1256, id526, id1189) belong to Cluster 2. Cluster 2 also includes two strains (id285 and id257) from an unknown serogroup exhibiting Sarmin/Pyrogenes/Javanica-like patterns. Cluster 3 comprises all *L. santarosai* strains belonging to serogroups Shermani (id96, id261, id262, id1304, id1252, id1255) and Tarassovi (id266, id289, id1295, id1303, id1467, id1254, id1214, id1261), along with strains of unknown serogroups (id1323, id1331, id282, id1320, id1324) showing patterns related to serogroups Tarassovi and Shermani and one strain (id944) with many *rfb* genes not present in other genomes, suggesting that it may belong to a new serovar/serogroup or a serovar/serogroup not included in our analysis. Finally, strains from cluster 4 belong to the antigenically related serogroups Mini, Hebdomadis and Sejroe which share a similar *rfb* gene composition [3]. *L. santarosai* strains from cluster 4 share similar genetic fingerprints and belong to serogroups Mini (id1325, id1319, id219, id89, id88), Sejroe (id1327, id1285, id1284), and Hebdomadis (id1251, id1322) [12], in addition to strains of unknown serogroups (id1309, id221, id1353, id281, id283, id1326) and strains id290 and id291 with more variability of genetic fingerprints.

Our analysis of the gene composition of the *rfb* cluster is consistent with previous studies showing that *L. santarosai* only contains serovars from serogroups Shermani, Hebdomadis, Tarassovi, Pyrogenes, Autumnalis, Bataviae, Mini, Grippotyphosa, Sejroe, Pomona, Javanica, and Sarmin [49], as well as probable new serovars. However, it must be noted that most of the *L. santarosai* genomic assemblies analyzed here are fragmented (average contig number, 149), which could lead to inaccurate serotype assignations. Further studies should include less fragmented genomes, ideally closed genomes, for a correct interpretation of the *rfb* patterns and to better identify the genes determining the serovar identity. As previously shown [1] most serogroups had a polyphyletic distribution. Thus, isolates from the serogroups Tarassovi and Grippotyphosa did not all cluster together in the phylogenetic tree based on either cgMLST alleles (**Fig 4**) or core-genes (**S5 Fig**).

## Conclusion

In conclusion, genome analyses showed species-specific genome size and GC content and an open pangenome in pathogenic species, with the exception of *L. mayottensis*. Taken together, these analyses suggest an ancient speciation of pathogens and their adaptation to diverse niches resulting in a great genotypic and phenotypic diversity across species. We also showed that despite the limited geographic distribution of *L. santarosai* to America, this species exhibits great diversity and an open pangenome. This study represents the largest and most detailed analysis of the genetic and serotype diversity of this pathogen to date, thus providing a comprehensive analysis of this pathogenic species. Our collection of *L. santarosai* exhibits an overrepresentation of isolates from America and more genomes representing undersampled regions and different animal reservoirs will be necessary to better understand the evolutionary history, epidemiology, and population dynamics of *L. santarosai*. We discovered a large genetic diversity among isolates from both human and animal samples, with no apparent transmission from one host to another, although circulation of strains that share the same serogroup was evident in multiple hosts. Outbreak investigations performed at the local level would likely improve the identification of animal reservoirs. These results will improve our understanding of the dissemination of genotypes in specific geographic regions and update the knowledge of strains circulating in America for effective disease surveillance.

## Supporting information

S1 Table Geographic origins of the most frequent pathogenic *Leptospira* species in our genome database (n = 914). (XLSX)

S2 Table List of *L. santarosai* strains used in this study and genome characteristics. (XLSX)

S3 Table Dunn Kruskal-Wallis multiple comparisons results between the G+C% content of *Leptospira* genomes. (DOCX)

S4 Table Dunn Kruskal-Wallis multiple comparisons results between the genome size (length) of *Leptospira* genomes. (DOCX)

S1 Fig Average Nucleotide Identity (ANI) among *L. santarosai* strains. *L. interrogans* strain Fiocruz L1-130 and *L. borgpetersenii* strain M84 were included as outgroups. The ANI percentages are depicted as colors of the square matrix elements, according to the scale shown in the upper left insert. The names of the 64 *L. santarosai* strains are indicated on the right of the matrix. Clustering shown on the left side (and upper side, symmetric) of the matrix table, was performed by GET_HOMOLOGUES version 20190411.
(PDF)

S2 Fig Percentage of Conserved Proteins (POCP) among *L.santarosai* strains. *L. interrogans* strain Fiocruz L1-130 and *L. borgpetersenii* strain M84 were included as reference of distinct species. POCP percentages are represented as colors of the square matrix elements, according to the scale shown in the upper left insert. The names of the 64 *L. santarosai* strains are indicated on the right of the matrix. Clustering shown on the left side (and upper side, symmetric) of the matrix table, was performed by GET_HOMOLOGUES version 20190411.
(PDF)

S3 Fig Pangenome analysis of *L.santarosai* strains. The graph was obtained with Roary 3.11.2 (yielding a total of 13,168 gene clusters). The pangenome of *L. santarosai* presents an open profile, which was further verified by Heap’s law [3], n = κNγ; considering a total of 13,168 genes (n) in the pangenome (according to Roary) and the 64 genomes (N) included, the observed curve allows for non-linear fitting with a constant κ = 2851.4. Thus, 1-γ = α = 0.63. An α value <1 indicates an open profile. Same result was obtained considering n = 13,156 (GET_HOMOLOGUES).
(PDF)

S4 Fig Jaccard similarity matrix for the comparative analyses of the *rfb* clusters. Jaccard similarity matrix is organized according to the gene presence/absence matrix of *rfb* clusters shown in Fig 5. To perform the Jaccard similarity index calculation, each strain (column) was converted into a vector of 0 (gene absence) and 1 (gene presence), and then used to do pairwise comparisons between the vectors using a Python pipeline via NumPy. Jaccard similarity is represented in colors, according to the scale shown in the lower right insert.
(PDF)

S5 Fig Core- genome based phylogeny of *L.santarosai*. Phylogenetic tree based on the sequences of 1288 core-genes of *L. santarosai*. A core-gene alignment was obtained by Roary (60% identity cut-off), and then used to perform the phylogeny. The best-fit model and the maximum-likelihood phylogenetic tree were determined by IQ-TREE version 1.6.11 [5], considering 10,000 ultrafast bootstraps [6]. *L. interrogans* strain Fiocruz L1-130 and *L. borgpetersenii* strain M84 were included as outgroups. The serogroup of each strain is indicated in parenthesis, as well as the world region (AF = Africa; CA = Central America; CB = Caribbean; NA = North America; SA = South America; SAs = South Asia). Bootstraps values other than 100% are shown.
(PDF)
