# Supplementary material for: Phylogenomics of Leptospira santarosai, a prevalent pathogenic species in the Americas
Source: PLoS Negl Trop Dis. 2023 Nov 2;17(11):e0011733. doi: 10.1371/journal.pntd.0011733 (PMC10645364; doi:10.1371/journal.pntd.0011733)
Supplement: S3 Table — (DOCX) [file pntd.0011733.s003.docx]

**Supp. Table 3.**  Dunn Kruskal-Wallis multiple comparisons results between the G+C% content of *Leptospira* genomes.

| **Genome 1** | **Genome 2** | **Z** | **P unadjusted** | **P adjusted*** | **significance?** |
| --- | --- | --- | --- | --- | --- |
| *L. borgpetersenii* | *L. interrogans* | 23.695601 | 4.00E-124 | **8.405E-123** | **yes** |
| *L. borgpetersenii* | *L. kirschneri* | 6.7034072 | 2.04E-11 | **4.2759E-10** | **yes** |
| *L. interrogans* | *L. kirschneri* | -8.8324078 | 1.02E-18 | **2.1514E-17** | **yes** |
| *L. borgpetersenii* | *L. mayottenensis* | 3.0745397 | 2.11E-03 | **0.04427381** | **yes** |
| *L. interrogans* | *L. mayottenensis* | -7.1513896 | 8.59E-13 | **1.804E-11** | **yes** |
| *L. kirschneri* | *L. mayottenensis* | -1.2599567 | 2.08E-01 | 1.00 | ns |
| *L. borgpetersenii* | *L. noguchii* | 5.4434492 | 5.23E-08 | **1.0974E-06** | **yes** |
| *L. interrogans* | *L. noguchii* | -4.4448081 | 8.80E-06 | **0.00018474** | **yes** |
| *L. kirschneri* | *L. noguchii* | 0.998442 | 3.18E-01 | 1.00 | ns |
| *L. mayottenensis* | *L. noguchii* | 1.8619713 | 6.26E-02 | 1.00 | ns |
| *L. borgpetersenii* | *L. santarosai* | -5.0288379 | 4.93E-07 | **1.0363E-05** | **yes** |
| *L. interrogans* | *L. santarosai* | -19.0799017 | 3.71E-81 | **7.7901E-80** | **yes** |
| *L. kirschneri* | *L. santarosai* | -9.2837431 | 1.64E-20 | **3.4362E-19** | **yes** |
| *L. mayottenensis* | *L. santarosai* | -5.9165522 | 3.29E-09 | **6.904E-08** | **yes** |
| *L. noguchii* | *L. santarosai* | -7.9228156 | 2.32E-15 | **4.876E-14** | **yes** |
| *L. borgpetersenii* | *L. weilii* | -2.3345431 | 1.96E-02 | 0.41 | ns |
| *L. interrogans* | *L. weilii* | -11.2352603 | 2.74E-29 | **5.7474E-28** | **yes** |
| *L. kirschneri* | *L. weilii* | -5.7424584 | 9.33E-09 | **1.9595E-07** | **yes** |
| *L. mayottenensis* | *L. weilii* | -3.9706147 | 7.17E-05 | **0.00150544** | **yes** |
| *L. noguchii* | *L. weilii* | -5.630652 | 1.80E-08 | **3.7701E-07** | **yes** |
| *L. santarosai* | *L. weilii* | 0.8471142 | 3.97E-01 | 1.00 | ns |

* p-values were adjusted with the Bonferroni method.
