# Supplementary material for: Phylogenomics of Leptospira santarosai, a prevalent pathogenic species in the Americas
Source: PLoS Negl Trop Dis. 2023 Nov 2;17(11):e0011733. doi: 10.1371/journal.pntd.0011733 (PMC10645364; doi:10.1371/journal.pntd.0011733)
Supplement: S4 Table — (DOCX) [file pntd.0011733.s004.docx]

**Supp. Table 4.**  Dunn Kruskal-Wallis multiple comparisons results between the genome size (length) of *Leptospira* genomes.

| **Genome 1** | **Genome 2** | **Z** | **P unadjusted** | **P adjusted*** | **significance?** |
| --- | --- | --- | --- | --- | --- |
| *L. borgpetersenii* | *L. interrogans* | -24.5110307 | 1.13E-132 | **2.37E-131** | **yes** |
| *L. borgpetersenii* | *L. kirschneri* | -10.6232542 | 2.32E-26 | **4.88E-25** | **yes** |
| *L. interrogans* | *L. kirschneri* | 5.266405 | 1.39E-07 | **2.92E-06** | **yes** |
| *L. borgpetersenii* | *L. mayottenensis* | -2.4309378 | 1.51E-02 | 0.32 | ns |
| *L. interrogans* | *L. mayottenensis* | 8.1625592 | 3.28E-16 | **6.89E-15** | **yes** |
| *L. kirschneri* | *L. mayottenensis* | 4.2046359 | 2.62E-05 | **5.49E-04** | **yes** |
| *L. borgpetersenii* | *L. noguchii* | -9.0038653 | 2.18E-19 | **4.58E-18** | **yes** |
| *L. interrogans* | *L. noguchii* | 1.1581012 | 2.47E-01 | 1.00 | ns |
| *L. kirschneri* | *L. noguchii* | -1.9252253 | 5.42E-02 | 1.00 | ns |
| *L. mayottenensis* | *L. noguchii* | -5.0390041 | 4.68E-07 | **9.83E-06** | **yes** |
| *L. borgpetersenii* | *L. santarosai* | -2.8806962 | 3.97E-03 | 0.08 | ns |
| *L. interrogans* | *L. santarosai* | 11.3498105 | 7.43E-30 | **1.56E-28** | **yes** |
| *L. kirschneri* | *L. santarosai* | 5.5136552 | 3.51E-08 | **7.38E-07** | **yes** |
| *L. mayottenensis* | *L. santarosai* | 0.2217722 | 8.24E-01 | 1.00 | ns |
| *L. noguchii* | *L. santarosai* | 5.9769778 | 2.27E-09 | **4.77E-08** | **yes** |
| *L. borgpetersenii* | *L. weilii* | -4.397792 | 1.09E-05 | **0.0002** | **yes** |
| *L. interrogans* | *L. weilii* | 4.7032591 | 2.56E-06 | **5.38E-05** | **yes** |
| *L. kirschneri* | *L. weilii* | 1.6047652 | 1.09E-01 | 1.00 | ns |
| *L. mayottenensis* | *L. weilii* | -1.8217257 | 6.85E-02 | 1.00 | ns |
| *L. noguchii* | *L. weilii* | 2.8380825 | 4.54E-03 | 0.10 | ns |
| *L. santarosai* | *L. weilii* | -2.2403587 | 2.51E-02 | 0.53 | ns |

* p-values were adjusted with the Bonferroni method.
