# Supplementary material for: Phylogenomics of Leptospira santarosai, a prevalent pathogenic species in the Americas
Source: PLoS Negl Trop Dis. 2023 Nov 2;17(11):e0011733. doi: 10.1371/journal.pntd.0011733 (PMC10645364; doi:10.1371/journal.pntd.0011733)
Supplement: S3 Fig — The graph was obtained with Roary 3.11.2 (yielding a total of 13,168 gene clusters). The pangenome of L. santarosai presents an open profile, which was further verified by Heap’s law [3], n = κNγ; considering a total of 13,168 genes (n) in the pangenome (according to Roary) and the 64 genomes (N) included, the observed curve allows for non-linear fitting with a constant κ = 2851.4. Thus, 1-γ = α = 0.63. An α value <1 indicates an open profile. Same result was obtained considering n = 13,156 (GET_HOMOLOGUES). (PDF) [file pntd.0011733.s007.pdf]

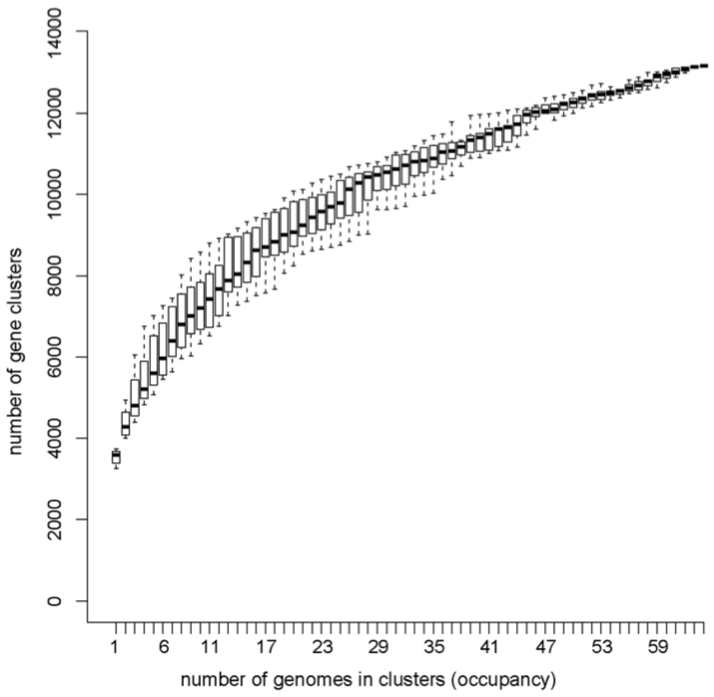

Heap's law parameter α = 0.63

S3 Figure : Pangenome analysis of *L. santarosai* strains.
The graph was obtained with Roary 3.11.2 [1] (yielding a total of 13,168 gene clusters). The pangenome of *L. santarosai* presents an open profile, which was further verified by Heap's law [1], $n = \kappa N\gamma$; considering a total of 13,168 genes (n) in the pangenome (according to Roary) and the 64 genomes (N) included, the observed curve allows for non-linear fitting with a constant $\kappa = 2851.4$. Thus, $1\text{-}\gamma = \alpha = 0.63$. An α value <1 indicates an open profile. Same result was obtained considering n = 13,156 (GET_HOMOLOGUES).

References:
1. Page AJ, Cummins CA, Hunt M, Wong VK, Reuter S, Holden MTG, et al. Roary: rapid large-scale prokaryote pan genome analysis Bioinformatics. 2015;31:3691-83.
2. Tettelin H, Riley D, Cattuto C, Medini D. Comparative genomics: the bacterial pan-genome. Curr Opin Microbiol 2008;11:472-7.
