# Supplementary material for: Phylogenomics of Leptospira santarosai, a prevalent pathogenic species in the Americas
Source: PLoS Negl Trop Dis. 2023 Nov 2;17(11):e0011733. doi: 10.1371/journal.pntd.0011733 (PMC10645364; doi:10.1371/journal.pntd.0011733)
Supplement: S4 Fig — Jaccard similarity matrix is organized according to the gene presence/absence matrix of rfb clusters shown in Fig 5. To perform the Jaccard similarity index calculation, each strain (column) was converted into a vector of 0 (gene absence) and 1 (gene presence), and then used to do pairwise comparisons between the vectors using a Python pipeline via NumPy. Jaccard similarity is represented in colors, according to the scale shown in the lower right insert. (PDF) [file pntd.0011733.s008.pdf]

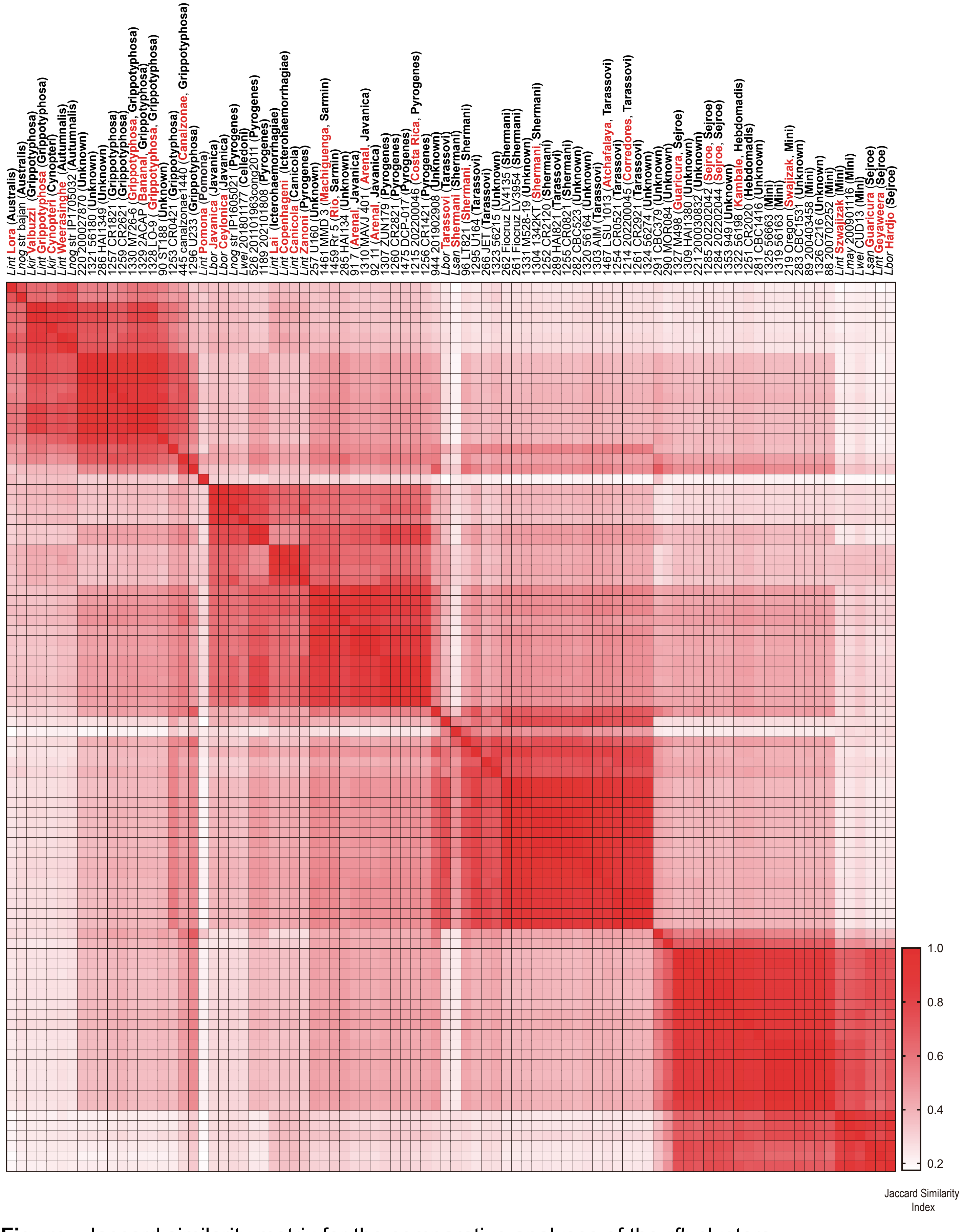

S4 Figure : Jaccard similarity matrix for the comparative analyses of the *rfb* clusters. Jaccard similarity matrix is organized according to the gene presence/absence matrix of *rfb* clusters shown in Figure 5. To perform the Jaccard similarity index calculation, each strain (column) was converted into a vector of 0 (gene absence) and 1 (gene presence), and then used to do pairwise comparisons between the vectors using a Python pipeline via NumPy [1]. Jaccard similarity is represented in colors, according to the scale shown in the lower right insert.

References:

1. Harris CR, Millman KJ, vanderWalt SJ, Gommers R, Virtanen P, Cournapeau D, et al. Array programming with NumPy. Nature. 385:357–62.
