# Supplementary material for: Phylogenomics of Leptospira santarosai, a prevalent pathogenic species in the Americas
Source: PLoS Negl Trop Dis. 2023 Nov 2;17(11):e0011733. doi: 10.1371/journal.pntd.0011733 (PMC10645364; doi:10.1371/journal.pntd.0011733)
Supplement: S5 Fig — Phylogenetic tree based on the sequences of 1288 core-genes of L. santarosai. A core-gene alignment was obtained by Roary (60% identity cut-off), and then used to perform the phylogeny. The best-fit model and the maximum-likelihood phylogenetic tree were determined by IQ-TREE version 1.6.11 [5], considering 10,000 ultrafast bootstraps [6]. L. interrogans strain Fiocruz L1-130 and L. borgpetersenii strain M84 were included as outgroups. The serogroup of each strain is indicated in parenthesis, as well as the world region (AF = Africa; CA = Central America; CB = Caribbean; NA = North America; SA = South America; SAs = South Asia). Bootstraps values other than 100% are shown. (PDF) [file pntd.0011733.s009.pdf]

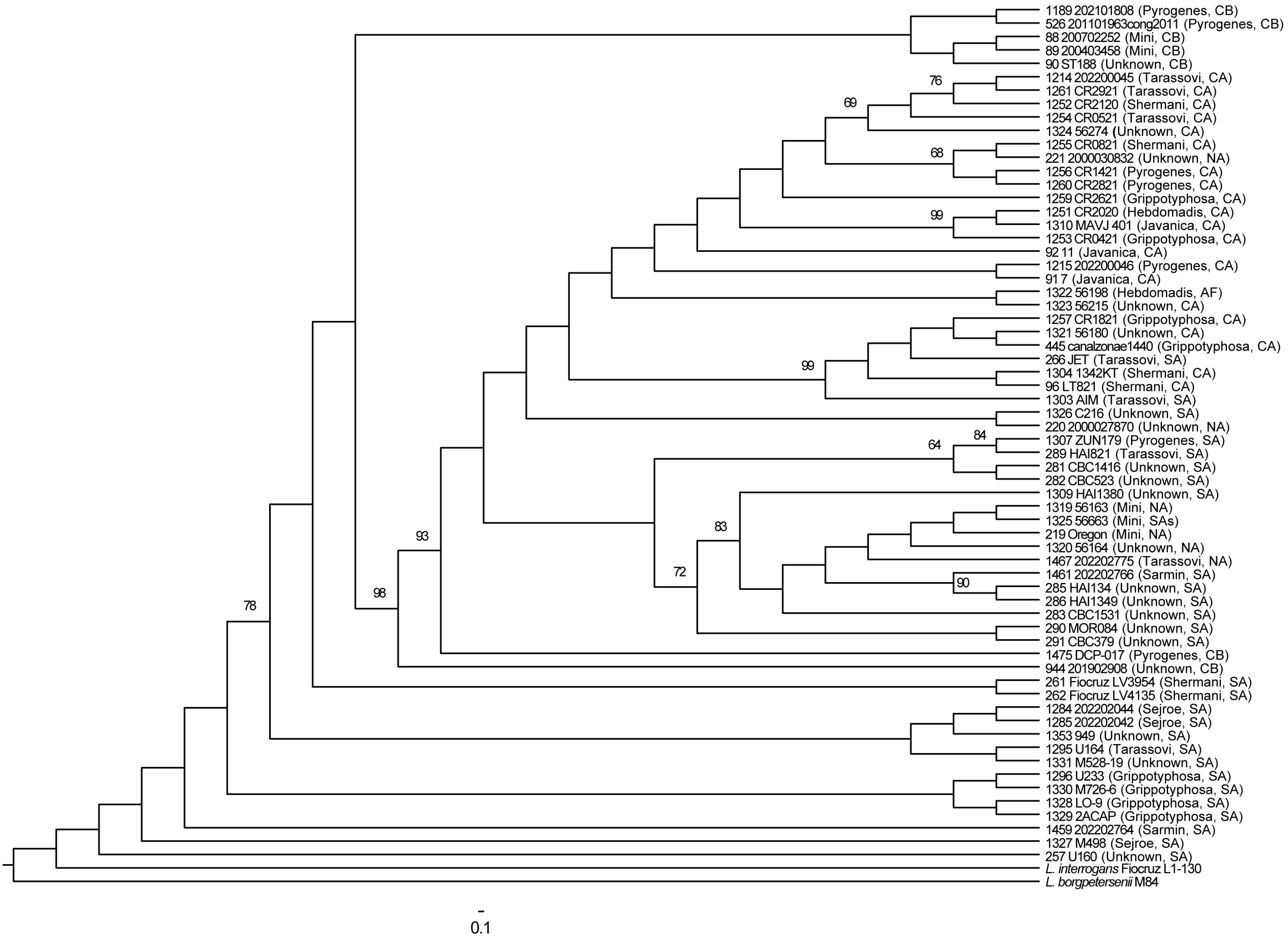

S5 Figure: Core- genome based phylogeny of *L. santarosai*. Phylogenetic tree based on the sequences of 1288 core-genes of *L. santarosai*. A core-gene alignment was obtained by Roary (60% identity cut-off), and then used to perform the phylogeny. The best-fit model and the maximum-likelihood phylogenetic tree were determined by IQ-TREE version 1.6.11 [5], considering 10,000 ultrafast bootstraps [6]. *L. interrogans* strain Fiocruz L1-130 and *L. borgpetersenii* strain M84 were included as outgroups. The serogroup of each strain is indicated in parenthesis, as well as the world region (AF=Africa; CA=Central America; CB=Caribbean; NA=North America; SA=South America; SAs=South Asia). Bootstraps values other than 100% are shown.

References:

1. Nguyen LT, Schmidt HA, vonHaeseler A, Minh BQ. IQ-TREE: a fast and effective stochastic algorithm for estimating maximum-likelihood phylogenies. Mol Biol Evol 2015;32:268-74.
2. Hoang DT, Chernomor O, vonHaeseler A, Minh BQ, Vinh LS. UFBoot2: Improving the Ultrafast Bootstrap Approximation. Mol Biol Evol. 2018;35:518-22.
