# Supplementary material for: Phylogenomics of Leptospira santarosai, a prevalent pathogenic species in the Americas
Source: PLoS Negl Trop Dis. 2023 Nov 2;17(11):e0011733. doi: 10.1371/journal.pntd.0011733 (PMC10645364; doi:10.1371/journal.pntd.0011733)
Supplement: S2 Fig — L. interrogans strain Fiocruz L1-130 and L. borgpetersenii strain M84 were included as reference of distinct species. POCP percentages are represented as colors of the square matrix elements, according to the scale shown in the upper left insert. The names of the 64 L. santarosai strains are indicated on the right of the matrix. Clustering shown on the left side (and upper side, symmetric) of the matrix table, was performed by GET_HOMOLOGUES version 20190411. (PDF) [file pntd.0011733.s006.pdf]

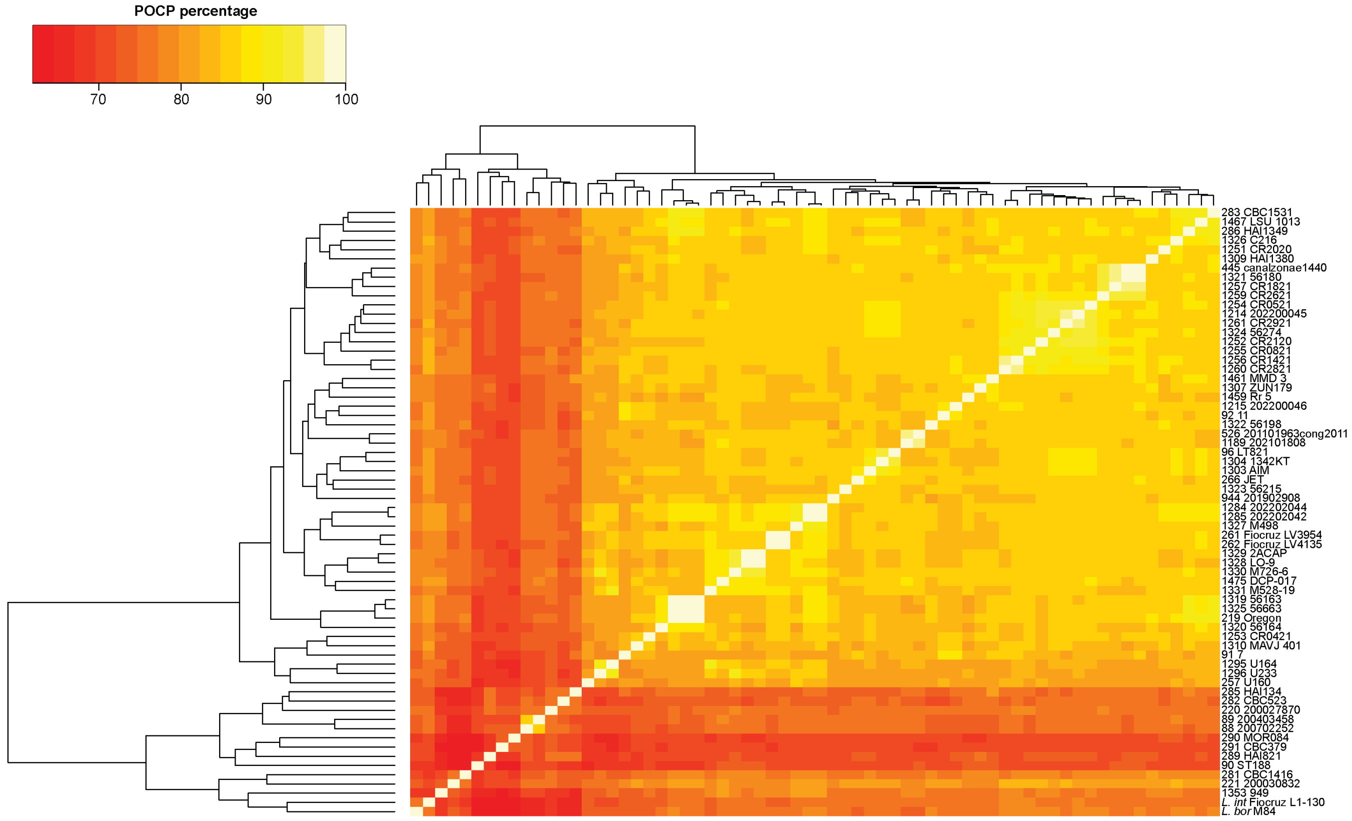

S2 Figure: Percentage of Conserved Proteins (POCP) among *L. santarosai* strains. *L. interrogans* strain Fiocruz L1-130 and *L. borgpetersenii* strain M84 were included as reference of distinct species. POCP percentages are represented as colors of the square matrix elements, according to the scale shown in the upper left insert. The names of the 64 *L. santarosai* strains are are indicated on the right of the matrix. Clustering shown on the left side (and upper side, symmetric) of the matrix table, was performed by GET_HOMOLOGUES version 20190411 [1].

References:

1. Contreras-Moreira B, Vinuesa P. GET_HOMOLOGUES, a versatile software package for scalable and robust microbial pangenome analysis. Appl Environ Microbiol. 2013;79:7696-701.
